# Parent and caregiver perceptions about the safety and effectiveness of foreign and domestic vaccines in Shanghai, China

**DOI:** 10.1371/journal.pone.0197437

**Published:** 2018-05-21

**Authors:** Zhuoying Huang, Xiaodong Sun, Abram L. Wagner, Jia Ren, Matthew L. Boulton, Lisa A. Prosser, Brian J. Zikmund-Fisher

**Affiliations:** 1 Department of Immunization Program, Shanghai Municipal Centers for Disease Control & Prevention, Shanghai, China; 2 Department of Epidemiology, School of Public Health, University of Michigan, Ann Arbor, MI, United States of America; 3 Department of Internal Medicine, Division of Infectious Diseases, University of Michigan Medical School, Ann Arbor, MI, United States of America; 4 Child Health Evaluation and Research Unit, Department of Pediatrics and Communicable Diseases, University of Michigan Medical School, Ann Arbor, MI, United States of America; 5 Department of Health Behavior & Health Education, School of Public Health, University of Michigan, Ann Arbor, MI, United States of America; 6 Department of Internal Medicine, Division of General Medicine, University of Michigan Medical School, Ann Arbor, MI, United States of America; La Trobe University, AUSTRALIA

## Abstract

**Background:**

Chinese parents have access to domestic and foreign vaccines for their children. Their vaccine preferences are unclear, especially given recent pharmaceutical quality scandals and widely held beliefs deriving from Traditional Chinese Medicine (TCM). This study characterized parental beliefs about the safety and effectiveness of Chinese and foreign vaccines.

**Methods:**

In May 2014, caregivers of young children at public immunization clinics in Shanghai, China, responded to a survey on vaccine perceptions. The two outcomes (differential belief in the effectiveness and safety of foreign vs domestic vaccines) were separately regressed onto demographic predictors in multinomial logistic regression models.

**Results:**

Among 618 caregivers, 56% thought the effectiveness of domestic and foreign vaccines were comparable; 33% thought domestic were more effective and 11% foreign. Two-thirds thought foreign and domestic vaccines had similar safety; 11% thought domestic were safer and 21% thought foreign were safer. Compared to college graduates, those with a high school education or less had greater odds of believing domestic vaccines were more effective, and also had greater odds of believing imported vaccines were safer. Greater trust in TCM was not associated with differential beliefs in the effectiveness or safety of domestic vs foreign vaccines.

**Conclusions:**

Although there is no evidence that foreign and domestic vaccines differ in either effectiveness or safety, less educated caregivers in China (but not those with greater trust in TCM) appear to believe such differences exist. Further exploration of the causes of these beliefs may be necessary in order to optimize vaccine communications in China.

## Introduction

Vaccination are an important part of infectious disease control efforts in China [1,2]. China has a two-tiered vaccination program. The government provides category 1 vaccines for free to citizens throughout the country at immunization clinics and category 2 vaccines are available for a cost at these same clinics. The China Experts Advisory Committee on Immunization Program decides which vaccines are category 1 vaccines within the Expanded Program on Immunization (EPI) [3]. The EPI in China started in 1978 with Bacillus Calmette-Guérin vaccine, oral polio vaccine, measles vaccine, and diphtheria-tetanus-pertussis vaccine, and it has since expanded to include hepatitis B vaccine in 2002, as well as hepatitis A, rubella, mumps, meningococcal meningitis, and Japanese encephalitis in 2007 [3].

Both domestic, Chinese vaccines and foreign, imported vaccines are available in China. Category 1 vaccines are produced by Chinese manufacturers [4], but EPI vaccines produced by foreign manufacturers are also available for sale at immunization clinics and are considered category 2 vaccines. After a clinical trial, and subsequent approval from the China Food and Drug Administration, other vaccines can be sold and administered at immunization clinics as category 2 vaccines [5]. Category 2 vaccines include those produced by both domestic and foreign manufacturers (pneumococcal polysaccharide vaccine, influenza vaccine, and *Haemophilus influenzae* type b vaccine), by only domestic manufacturers (enterovirus 71 vaccine, rotavirus vaccine and cholera vaccine), and by only foreign manufacturers (pneumococcal conjugate vaccine and human papillomavirus vaccine). Because of their cost and because they are not required for school entry, category 2 vaccines have lower coverage than category 1 vaccines; these vaccines are also often administered to infants and children who are older [6], and past the age at which infants may be most vulnerable to serious disease. Nonetheless, in Shanghai, category 2 vaccines are a substantial proportion of all doses administered– 46.3% in 2017 (Personal Communication, Xiang Guo, Shanghai CDC, 6 April 2018).

Studies that have directly compared domestic and imported vaccines in China have found similar immunogenicity and safety profiles. In a study of a domestically produced and imported measles-mumps-rubella vaccine (MMR), the proportion of individuals seropositive after vaccination with either vaccine was within three percentage points for measles and rubella (a larger spread of seven percentage points was seen for mumps), and the rate of adverse events following immunization was similar for the two vaccines (3.92/100,000 doses administered for the domestic vaccine and 1.39/100,000 for the imported vaccine) [7].

Little information is available on differential parental perceptions of vaccines from China vs other countries. Beliefs about safety and effectiveness could be influenced by concern about the quality of pharmaceutical products originating within China. Recent news articles have highlighted how counterfeit products have been linked to serious illness in children and hospital patients [8–10].

An additional but competing influence on vaccine decision-making in China could be concepts from Traditional Chinese Medicine (TCM). Currently, 12% of licensed doctors in China are TCM practitioners [11], and the Chinese government has officially supported the promotion and development of TCM [12]. TCM includes concepts like body constitution, which describes an individual’s susceptibility to certain diseases and can better facilitate personalizing health prevention and promotion activities [13]. Therefore, beliefs about pediatric vaccines could be influenced by a family’s consideration of quality of Chinese vs foreign-made products, as well as the suitability for products made in China vs other countries for the body of a Chinese child according to TCM considerations. Qualitative [14] and quantitative [15] studies of parents in Australia have found a relationship between greater beliefs in TCM and vaccination skepticism; although similar studies have not been conducted in China, beliefs about TCM could plausibly affect beliefs about vaccines, in general, and in imported vaccines, in particular.

We conducted a study to characterize caregivers’ differential beliefs about the safety and effectiveness of Chinese vs imported vaccines in Shanghai, China. Secondarily, we wanted to assess the relationship between trust in TCM and Western medicine and differential beliefs about Chinese vs imported vaccines. These relationships can be used to better understand how parents in China develop beliefs about vaccines and can be a starting point for thinking about how best to develop vaccine messaging that targets parents’ attitudes, assumptions, and scientific understanding.

## Methods

### Study population

This cross-sectional study enrolled caregivers (i.e., mothers, fathers, or other) of children aged 8 months to 7 years in May and June of 2014. We employed a two-stage cluster sampling, with township immunization clinics as the clusters. Townships were selected by a probability proportionate to size systematic selection procedure based on the population listed in the China 2010 Census. Within each clinic, we selected a convenience sample of 20 caregivers. The sample size calculation was based on the aims of another project (comparing measles vaccination timeliness of 81% in non-locals and 91% in locals, which required a total simple random sample size of 416). We assumed an intracluster correlation coefficient of 0.024 based on a previous study [16]. With 20 people sampled per cluster, the design effect was 1.456, and the effective sample size was 606.

### Study measures

The questionnaire collected information on caregiver perceptions of pediatric vaccines. Many questions were on a 5-point Likert scale from 1 to 5. Perceptions of vaccine effectiveness was measured by the question “How effective are [foreign-made | Chinese-made] vaccines in Chinese children? (selected from “(1) Not at all effective” to “(5) Extremely effective”). Perceived vaccine safety corresponded to the question “How safe are [foreign-made | Chinese-made] vaccines in Chinese children? (“(1) Not at all safe” to “(5) Extremely safe”). We also measured perceptions of different medicine systems (beyond vaccinations) through the question “How trustworthy is [TCM | Western medicine] to cure infectious disease?” (“(1) Not at all trustworthy” to “(5) Extremely trustworthy”). Participants were also asked: “How trustworthy are recommendations from your doctors at the immunization clinic?” on the same scale. The questionnaire is available as part of a previous publication [17].

Both outcomes (differential belief in effectiveness of domestic vs imported vaccines and differential belief in safety of domestic vs imported vaccines) were created by placing participants into one of three categories: those who believed that foreign and domestic vaccines had the same safety (or effectiveness), those who believed that foreign vaccines were safer (or more effective), and those who believed that domestic vaccines were safer (or more effective).

### Statistical analysis

The distribution of the main variables of interest are described with proportions or means and standard errors (SE). The correlations among beliefs were assessed with Spearman’s coefficient.

Both outcomes were separately regressed onto demographic predictors using a multinomial logistic regression model, where the referent category was belief that imported and domestic vaccines had the same effectiveness or safety. Significance was assessed at an α level of 0.05, and precision of results evaluated through 95% confidence intervals (CI). Survey procedures were used in the analysis, including clustering at the township level and using sampling weights. All analyses were conducted in SAS version 9.4 (SAS Institute, Cary, NC, USA).

### Ethical statement

This study was approved by the University of Michigan Health Sciences and Behavioral Sciences Institutional Review Board (#HUM00087564) and the Shanghai CDC Ethical Review Committee (#2014–10). Verbal informed consent was obtained from all individual participants included in the study prior to data collection.

## Results

Of 734 caregivers of children approached at immunization clinics, 618 (84%) agreed to participate in the survey. Most participants (65%) were mothers, the remainder were fathers (28%) or other family members (8%), usually grandmothers (Table 1). The participants were balanced between local (44%) vs non-local (56%) residents.

**Table 1:** Demographic distribution of study participants in Shanghai, China.

		Unweighted count	Weighted % (95% CI)
Relationship to child	Mother	405	65% (60%, 69%)
	Father	156	28% (24%, 31%)
	Other	57	8% (4%, 11%)
Sex of child	Male	324	51% (46%, 57%)
	Female	292	49% (43%, 54%)
Age of child	8 to <18 months	163	27% (24%, 30%)
	18 months to <2 years	138	22% (18%, 26%)
	2 to <3 years	117	20% (15%, 24%)
	3 to <4 years	78	13% (9%, 16%)
	4 to <7 years	122	19% (14%, 23%)
Residency	Local	312	44% (36%, 51%)
	Non-local	304	56% (49%, 64%)
Education	High school or less	235	42% (34%, 50%)
	Some post-secondary education	153	23% (19%, 27%)
	College graduate	227	35% (27%, 42%)
Monthly family income	<6,000 RMB	251	43% (35%, 51%)
	6 to <10,000 RMB	156	27% (21%, 32%)
	≥10,000 RMB	208	30% (23%, 38%)

Notes: CI, confidence interval

A majority of respondents (357, 56%) thought the effectiveness of domestic and imported vaccines were comparable, whereas 182 (33%) thought domestic were more effective and 76 (11%) foreign. Two-thirds (415, 67%) thought foreign and domestic vaccines had similar safety, whereas 11% (75) thought domestic were safer and 21% (124) foreign. Trustworthiness of Western medicine to treat infectious disease was 3.76, compared to 3.28 for TCM. About half (319, 54%) thought Western medicine and TCM were similarly trustworthy; 35 (6%) thought TCM more trustworthy and 261 (40%) thought Western medicine more trustworthy.

Participants average rating of trust in doctors was 4.11 (Table 2). Correlations between variables are shown in Table 1. Beliefs about the effectiveness and safety of imported and domestic vaccines were highly correlated; although the correlations were higher between the two questions about imported vaccines and the two questions about domestic vaccines than they were between the domestic vaccines and imported vaccines. Beliefs about the trustworthiness of Western medicine and TCM were also significantly, positively correlated.

**Table 2:** Beliefs about infectious disease medicine in Shanghai, China.

			Spearman’s Correlation Coefficient						
	Mean	SE	A.	B.	C.	D.	E.	F.	G.
A. Effectiveness of foreign vaccines	3.72	0.06	1.00	0.44***	0.63***	0.38***	0.26***	0.09*	0.22***
B. Effectiveness of domestic vaccines	3.98	0.05		1.00	0.37***	0.58***	0.21***	0.14**	0.25***
C. Safety of foreign vaccines	3.73	0.07			1.00	0.58***	0.22***	0.07	0.21***
D. Safety of domestic vaccines	3.85	0.05				1.00	0.18***	0.07	0.26***
E. Trust in Western medicine	3.76	0.05					1.00	0.35***	0.17***
F. Trust in Traditional Chinese Medicine	3.28	0.04						1.00	0.08*
G. Trust in doctors	4.11	0.04							1.00

Notes: SE, standard error

* P<0.05

** P<0.01

*** P<0.0001

Table 3 lists the regression results. Income and education were significantly related to beliefs about vaccine safety and effectiveness. Compared to college graduates, individuals with a high school education or less had 2.63 times higher odds of believing domestic vaccines were more effective (95% CI: 1.25, 5.52), and also had 3.69 times higher odds of believing that imported vaccines were safer (95% CI: 1.64, 8.30). For income, compared to individuals in families with the highest income levels, individuals whose families made 6 to <10,000 RMB per month had 0.52 times the odds of believing imported vaccines were safer (95% CI: 0.29, 0.93). As individuals expressed greater trust in doctors, the odds of believing imported vaccines were safer increased 1.28 times (95% CI: 1.03, 1.59). Higher levels of trust in Western medicine or TCM was not associated with differential beliefs in the effectiveness or safety of domestic vs imported vaccines.

**Table 3:** Differences in beliefs about domestic and foreign vaccines, and Western and Traditional Chinese Medicine in Shanghai, China.

	Vaccine effectiveness				Vaccine safety			
	Domestic more effective		Foreign more effective		Domestic safer		Foreign safer	
	OR (95% CI)	P	OR (95% CI)	P	OR (95% CI)	P	OR (95% CI)	P
**Relationship**								
Mother	ref		ref		ref		ref	
Father	1.47 (0.96, 2.24)	0.0718	0.95 (0.46, 1.96)	0.8882	0.91 (0.46, 1.81)	0.7789	0.93 (0.60, 1.46)	0.7563
Other	0.78 (0.34, 1.78)	0.5421	0.46 (0.11, 1.96)	0.2821	0.57 (0.14, 2.36)	0.4224	0.80 (0.26, 2.41)	0.6780
**Female vs male child**	0.85 (0.49, 1.49)	0.5640	0.42 (0.18, 0.95)	0.0384	0.72 (0.35, 1.50)	0.3704	1.21 (0.81, 1.81)	0.3364
**Age of child**								
8 to <18 months	ref		ref		ref		ref	
18 months to <2 years	0.77 (0.46, 1.28)	0.3041	1.75 (0.71, 4.26)	0.2126	1.21 (0.51, 2.82)	0.6583	1.22 (0.63, 2.37)	0.5397
2 to <3 years	0.71 (0.27, 1.85)	0.4637	0.62 (0.23, 1.69)	0.3408	0.68 (0.23, 2.02)	0.47	1.24 (0.61, 2.53)	0.5346
3 to <4 years	0.95 (0.40, 2.28)	0.9111	1.01 (0.31, 3.35)	0.9806	0.72 (0.23, 2.29)	0.5703	1.14 (0.37, 3.56)	0.8105
4 to <7 years	0.64 (0.29, 1.42)	0.2658	0.73 (0.26, 2.08)	0.5454	1.34 (0.45, 4.02)	0.5898	0.96 (0.52, 1.74)	0.8792
**Non-local vs local**	1.18 (0.67, 2.07)	0.5527	0.53 (0.21, 1.38)	0.1861	0.47 (0.20, 1.09)	0.0774	1.10 (0.56, 2.15)	0.7711
**Education**								
High school or less	2.63 (1.25, 5.52)	0.0123	1.16 (0.41, 3.27)	0.7702	1.09 (0.36, 3.25)	0.8809	3.69 (1.64, 8.30)	0.0026
Some post-secondary education	1.94 (0.87, 4.30)	0.1012	1.47 (0.68, 3.17)	0.3171	1.30 (0.65, 2.60)	0.4444	1.97 (0.78, 4.98)	0.1476
College graduate	ref		ref		ref		ref	
**Monthly family income**								
<6,000 RMB	1.18 (0.46, 3.01)	0.7186	0.94 (0.37, 2.36)	0.8907	0.67 (0.32, 1.40)	0.2731	0.82 (0.35, 1.88)	0.6204
6 to <10,000 RMB	0.98 (0.33, 2.86)	0.9655	0.63 (0.23, 1.70)	0.3499	0.54 (0.26, 1.12)	0.0956	0.52 (0.29, 0.93)	0.0282
≥10,000 RMB	ref		ref		ref		ref	
**Trustworthiness of doctors**	1.00 (0.72, 1.39)	0.9958	1.12 (0.82, 1.54)	0.4584	1.06 (0.80, 1.40)	0.6844	1.28 (1.03, 1.59)	0.0299
**Trustworthiness of Western medicine**	1.00 (0.72, 1.39)	0.9835	0.89 (0.47, 1.69)	0.7114	1.14 (0.57, 2.28)	0.696	1.05 (0.79, 1.39)	0.7441
**Trustworthiness of Traditional Chinese Medicine**	0.96 (0.76, 1.21)	0.7106	1.10 (0.77, 1.57)	0.5893	0.98 (0.73, 1.31)	0.868	0.95 (0.70, 1.29)	0.7395

Notes: CI, confidence interval; OR, odds ratio; TCM, Traditional Chinese Medicine

## Discussion

Individuals who hold positive beliefs about vaccine safety and effectiveness are more likely to get vaccinated [18], and so understanding the origination of these beliefs in both Western and non-Western settings is important to preventing or minimizing vaccine hesitancy [19]. In this survey from Shanghai, China, most caregivers of young children rated domestic and foreign vaccines as having similar effectiveness and safety. Of those who expressed a difference between the two types of vaccines, slightly more believed that domestic vaccines were more effective than imported vaccines, but that foreign vaccines were safer than domestic vaccines.

This contrast that is seen in beliefs about effectiveness vs safety could be tied to Chinese caregivers’ confidence in domestic scientific enterprise vs regulatory agencies. Beliefs about effectiveness could be tied to beliefs about scientific capability (e.g., the clinical trials behind vaccine development): more Chinese individuals may have growing confidence that scientific enterprise in China is roughly equivalent in quality to that from abroad. Beliefs about safety might be more tied to the regulatory environment and the presence of recent food and medical scandals [8–10]. However, more research is needed on how beliefs about effectiveness and safety of medical interventions are formed.

Education was a strong factor that was related to differential beliefs in the effectiveness and safety of domestic vs imported vaccines. Interestingly, those with less education believed domestic vaccines more effective, but imported vaccines safer. It is unclear how these beliefs were formed, especially in low education groups. Most vaccine educational materials are tailored to a rational, analytical approach [20], and may be less effective in lower educational groups. Additionally, beliefs in both vaccine safety and effectiveness could undoubtedly be influenced by the vaccine scandal from spring 2016, when it was discovered that expired vaccines were improperly transported, possibly leading to a few deaths, and leading to many more children receiving ineffective vaccines [21]. Few studies have actually compared different vaccines. One study comparing an imported vs domestically-produced measles-mumps-rubella vaccine found immunogenicity was slightly higher in the Chinese-made vaccine (e.g., 86.76% seroconversion for mumps vs 79.17% for the foreign-made vaccine), and the rate of adverse events was relatively similar (3.92 per 100,000 population vs 1.39 per 100,000 population for the foreign-made vaccine) [7].

We found a moderate, but significant, positive correlation between trust in TCM and Western medicine. This could point to a distinction within the Chinese population among those with greater trust in health authorities (Traditional Chinese or Western) vs those with less trust in either. Beliefs about the effectiveness and safety of vaccines–both domestic and imported–more strongly correlated to trust in Western medicine than in TCM, although neither was related to a differential belief in the effectiveness or safety of domestic vs imported vaccines. Obviously the development of vaccines in China is an outgrowth of Western medicine, not TCM, so the correlations among beliefs about domestic vaccine safety, vaccine effectiveness, and Western medicine suggest that Chinese caregivers’ considerations of a “body constitution,” is more of a vague consideration of physical differences between Westerners and Chinese children, and not differences explicitly delineated by TCM. That greater trust in doctors was related to belief that imported vaccines were safer could point to the role of doctors in servicing as trusted sources of information. The important role of doctors in promoting vaccines has been extensively explored in the Western literature [22], and is particularly important in settings where caregivers and patients face complicated health care choices.

### Public health implications

There are a number of practical implications to our findings, beyond the emphasis that doctors serve as an important and highly trusted source of health information. Most vaccine messaging developed today is analytical and focused on facts [20]. However, health decision making is often based on heuristics and emotions that have little basis in evidence-based science; these heuristics or unconscious associations may be related to differential beliefs about imported vs. Chinese vaccines. For parents with a larger distrust of vaccines, generating narratives and providing consistent pro-vaccination messaging over time can sway opinions [20]. There is not any reason for vaccination providers to promote domestic or imported vaccines over the other, and high coverage of either can prevent the spread of infectious disease. However, certain vaccines, such as pneumococcal conjugate vaccine or human papillomavirus vaccine, will only be produced by foreign manufactures in the short term. Any attempts to promote coverage of these vaccines will require public health officials and vaccination providers to recognize that parents may have different beliefs and perceptions about domestic vs imported vaccines. Because caregivers tended to think domestic vaccines were more effective, but less safe than imported vaccines, vaccination providers could emphasize safety when promoting domestic vaccines and effectiveness when promoting imported vaccines.

### Strengths and limitations

There are several limitations. We examined the prevalence of certain beliefs, but cannot definitively establish causality, or even temporality. In addition, we are limited to the population of caregivers in Shanghai, and our study may not be generalizable to other locations in China whose populations differ by education and income. Because we sampled individuals from immunization clinics, there could be selection bias in that our study population may be more receptive to receiving immunization services than the general population. However, by sampling clinics throughout the city of Shanghai, we have a reasonable approximation of the diversity of beliefs within this city.

### Conclusions

Beliefs of caregivers of young children in China about vaccine safety and effectiveness were relatively positive in this cross-sectional survey, and most caregivers thought Chinese-made and imported vaccines had similar effectiveness and safety. As caregivers face choices about giving their children different vaccines, they are likely influenced by their educational background, and their understanding of different material, often presented to them by doctors. Trust in TCM was not associated with differential beliefs in vaccine safety or effectiveness of domestic vs imported vaccines. Further exploration of the causes of these beliefs may be necessary in order to optimize vaccine communications in China.
